# Incorporation of Magnetic Nanoparticles into Protoplasts of Microalgae *Haematococcus pluvialis*: A Tool for Biotechnological Applications

**DOI:** 10.3390/molecules25215068

**Published:** 2020-11-01

**Authors:** Maria G. Savvidou, Angelo Ferraro, Evangelos Hristoforou, Diomi Mamma, Dimitris Kekos, Fragiskos N. Kolisis

**Affiliations:** 1 Biotechnology Laboratory, School of Chemical Engineering, National Technical University of Athens, 9 Iroon Polytechniou Str, Zografou Campus, 15780 Athens, Greece; msavvid@central.ntua.gr (M.G.S.); an.ferraro2@gmail.com (A.F.); dmamma@chemeng.ntua.gr (D.M.); kekos@chemeng.ntua.gr (D.K.); 2 Laboratory of Electronic Sensors, School of Electrical and Computer Engineering, National Technical University of Athens, 9 Iroon Polytechniou Str, Zografou Campus, 15780 Athens, Greece; hristoforou@ece.ntua.gr

**Keywords:** magnetic nanoparticles, microalgae, *Haematococcus pluvialis*, protoplasts transformation, protoplasts regeneration, cellulose treatment

## Abstract

Intensive research on the use of magnetic nanoparticles for biotechnological applications of microalgae biomass guided the development of proper treatment to successfully incorporate them into these single-cell microorganisms. Protoplasts, as cells lacking a cell wall, are extensively used in plant/microalgae genetic manipulation as well as various biotechnological applications. In this work, a detailed study on the formation of protoplasts from *Haematococcus pluvialis* with the use of enzymatic and mechanical procedures was performed. The optimization of several parameters affecting the formation of protoplasmic cells and cell recovery was investigated. In the enzymatic treatment, a solution of cellulase was studied at different time points of incubation, whereas in the mechanical treatment, glass beads vortexing was used. Mechanical treatment gave better results in comparison to the enzymatic one. Concerning the cell recovery, after the protoplast formation, it was found to be similar in both methods used; cell viability was not investigated. To enhance the protoplast cell wall reconstruction, different “recovery media” with an organic source of carbon or nitrogen were used. Cell morphology during all treatments was evaluated by electron microscopy. The optimal conditions found for protoplast formation and cell reconstruction were successfully used to produce *Haematococcus pluvialis* cells with magnetic properties.

## 1. Introduction

Magnetic nanoparticles are a new trend in various scientific fields, such as drug delivery, DNA/RNA purification, improved magnetic resonance imaging (MRI), immobilization, food industry, medical diagnostics, cell harvesting, bioremediation, and others [1,2,3,4]. They are a class of particles consisting of a magnetic and a functional chemical component with a diameter varying from 1 to 100 nm and can be controlled by magnetic fields displaying superparamagnetism. *Haematococcus pluvialis* (*H. pluvialis*) is a microalgae strain rich in astaxanthin as well as lipids (20–25% per dry weight with 10% of it being polyunsaturated fatty acids), proteins (29–45% per dry weight), and carotenoids (2–5% per dry weight). Its ability to grow even in extreme conditions makes it a strategic tool for human diet and animal feeding and can be used even for anticancer and anti-inflammatory purposes [5]. Skin, heart and eye health, and photoprotection are also fields where *H. pluvialis* is an important implement [6]. 

Improved cultivation conditions that enhance the production of the above-mentioned high-value-added products as well as better harvesting methods are needed to fulfil the increased demands of *H. pluvialis* usage. Applications of nanomaterials in microalgae cultivation and harvesting are widely used for enhancing biomass and lipid production, reducing production costs, more efficient collection and purification, as well as the refinement process, giving the ability to reuse nanomaterials [7]. The use of magnetic nanoparticles, naked or surface functionalized iron oxide (Fe_3_O_4_) particles, has been reported in algal biomass harvesting as well as in biomedical applications [3,8,9,10]. Moreover, the generation of magnetically modified by the insertion of magnetic nanoparticles or with newly acquired genetic properties by the insertion of macromolecules bound to magnetic nanoparticles [11] into the microalgae protoplasts can provide new insights into microalgae biotechnological applications.

The microalgae cell wall is constituted by rigid components embedded in a polymeric matrix constituted by 70% cellulose as well as glycoproteins, pectin, and algaenan, which enhance the stiffness of their cell walls [12]. High-pressure homogenization following acidic and thermal pre-treatment, single enzymatic digestion with cellulase, hemicellulase, and pectinase, or a mix of them as well as combinatory methods of enzymatic and mechanical treatment (sonication and microwaves) are used for microalgae protoplast formation [13,14,15]. 

Protoplasts can be used as a convenient system to improve the delivery of nanocomposites or foreign DNA/RNA into microalgae. Nowadays, protoplast fusion of different species or organisms is a common technique for genome combination and development of microorganisms with desired properties as well as in biotechnological applications [14,16,17,18,19]. The extent of cell wall thickening, temperature and duration of the enzymatic incubation, pH, agitation, as well as the nature of osmotic solution are key factors for protoplast formation [13].

Protoplast isolation is also a complex task, especially when enzymatic digestion is used, which is a stress-induced procedure due to peroxisome production as well as degradation products that enhance cell lysis [20]. If necessary, flow cytometry sorting or high-gradient sucrose centrifugation is used for the separation of protoplasts from intact cells [21]. After protoplast isolation, the regeneration of the cell wall is of vital importance in order to fully exploit the genetic or biotechnological features that have been modified in the parental cells. Medium with a non-metabolizable sugar alcohol like mannitol or sorbitol, and with auxins and cytokinins, may provide protection against osmotic pressure and support mitosis and daughter cell formation [13]. Studies on *Chlorophyta* species after physical wounding demonstrate the recreation of a cell membrane on the surface of protoplasts 12 h after the wounding, while Golgi bodies with numerous vesicles at the peripheral region of the rebuilding cell at 24 h after the wounding began to develop [22]. 

*H. pluvialis*, a freshwater species of *Chlorophyta* of interest in many biotechnological applications, has not been studied in detail so far. Its cell wall has a different composition compared to other microalgae species [14]. In this work, a comparative study of the efficiency of enzymatic (cellulase) and mechanical (glass bead vortexing) treatments of *H. pluvialis* microalgae species targeting protoplasts formation and wall reconstruction, which is based on modified media enriched with different carbon and amino acids sources, was undertaken. Using scanning electron microscopy, the regeneration of the cell wall was analyzed; however, the survival rates were not investigated. Subsequently, iron oxide magnetic nanoparticles were used to test the efficiency of transformation via electroporation. Our findings provide important basic information on how to easily prepare and recover high-quantity *H. pluvialis* protoplasts for biotechnological applications.

## 2. Results

### 2.1. H. pluvialis Protoplasts Formation under Enzymatic or Mechanical Treatment

In order to accomplish our goal, the creation of transformable *H. pluvialis* microalgae, two different approaches for protoplast formation were used; the enzymatic, which is based on cellulase treatment, and the mechanical, which is based on glass bead vortexing.

**Figure 1:**
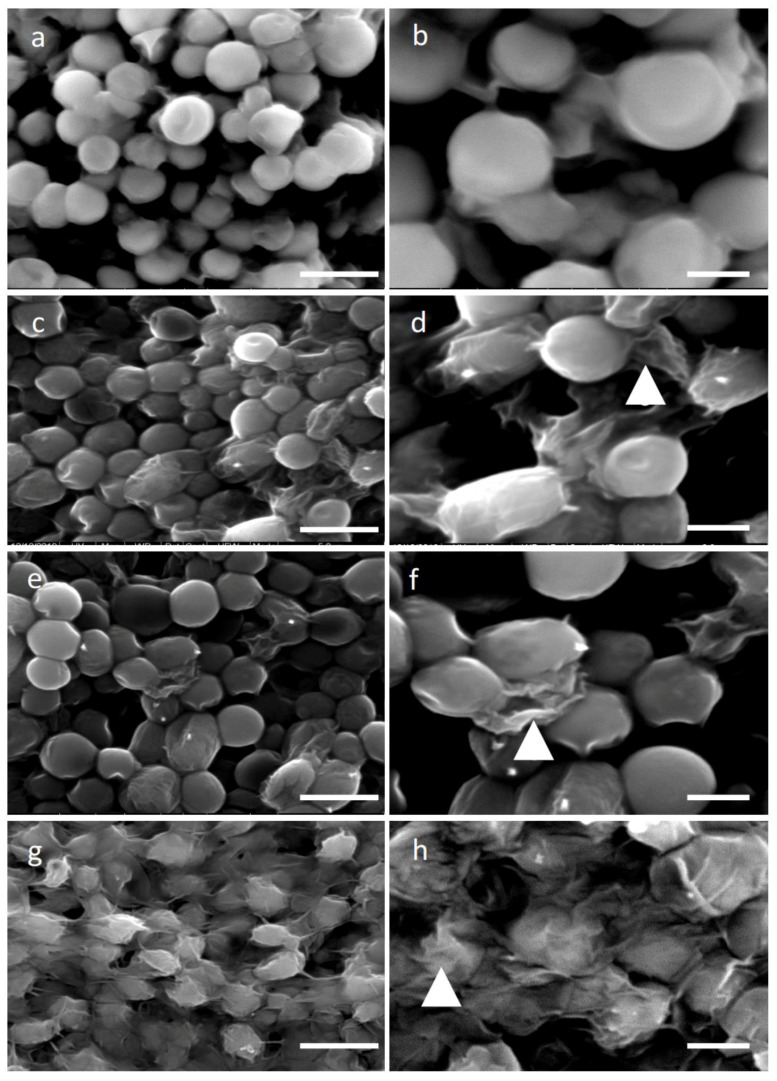
Scanning electron microscopy images of *H. pluvialis* cells before and after enzymatic/mechanical treatment, in two different magnifications (5000× left column, 10,000× right column) (**a**,**b**) SEM images of *H. pluvialis* cells with no treatment (control); (**c**,**d**) SEM images of *H. pluvialis* cell wall disruption with 4 h of enzymatic hydrolysis; (**e**,**f**) SEM images of *H. pluvialis* cell wall disruption with 16 h of enzymatic hydrolysis; (**g**,**h**) SEM images of *H. pluvialis* cell wall disruption after mechanical treatment with glass beads. Scale bars: 5 µm left column, 2 µm right column. White arrows in (**d**,**f**,**h**) indicate cell wall fragments.

Cellulose, hemicellulose, and pectin are the algae cell wall’s essential components. Cellulose dissociation results in degradation and thus becomes a more permeable and less rigid cell wall. Four different time points of 4, 8, 12, and 16 h at 30 °C of cellulase treatment (2%) were used in order to study the efficiency of *H. pluvialis* protoplast production. The concentration of 2% was chosen to limit the reaction cost. Scanning electron microscopy (SEM) images after 4 and 16 h of the enzymatic treatment allowed the study of the degree of cell wall damage. In Figure 1a,b, SEM images confirm the healthy and structure-complete microalgae cells without any cellulase treatment, while after 4 h of enzymatic digestion, partial disruption of the cell wall is evident (Figure 1c,d). Indeed, cellulase-treated cells are less spherical and more stretched compared to the round untreated cells and portions of the decomposed cell wall can be seen barely attached on top of the microalgae cells (arrows in Figure 1d,f,h). Furthermore, some cells appeared squeezed with the body structure completely collapsed, a sign that cellulase digestion caused significant damage, exacerbated by the fixation and vacuum treatments required for the preparation of SEM observation. After 16 h of cellulase treatment (Figure 1e,f), cells showed a similar morphology as compared to those digested for 4 h, a sign that confirms the treatment to be mild when a low enzyme concentration is used. 

Glass beads vortexing was used as a second method for protoplasts formation. Different proportions of glass beads (100, 200, and 300 mg in 5 mL) as well as vortexing time (15, 30, 60, 90, and 120 s) were tried, and all experiments were performed with the optimum conditions for 30 s and 200 mg of dry glass beads. Following mechanical treatment with glass beads, the altered morphology of the microalgae cell surface is shown in Figure 1g,h. The decomposed cell walls of protoplast cells are evident. SEM images reveal much more stressed cells as compared to those after the cellulase digestion, demonstrating a more severe phenotype. It seems that a big number of microalgae cells were affected by the mechanical process as compared to the enzymatic treatment.

### 2.2. Protoplast Recovery upon Carbon and Amino Acid Addition to the Culture Media

Following the protoplast preparation and the downstream treatments, the regeneration of the cell wall is a crucial procedure in order for the microalgae cells to be fully exploited. Due to treatment damages, we reasoned that for a fast cell wall reconstruction, the elementary cell wall components could not be synthesized based only on protoplast photosynthesis; therefore, extra carbon and amino acid sources were used to enhance metabolism and pathways related to the production of cellulose, phospholipids, and other cell wall components. Specifically, culture media supplemented with 1% and 2% *w*/*v* glucose, 1% and 2% *w*/*v* fructose, as well as 0.1% *w*/*v* casamino acids were tested. This approach gave the opportunity to evaluate five growth media to find the most efficient source of organic carbon or nitrogen for protoplast recovery after enzymatic or mechanical cell wall degradation. It was also noticed that the best way to recover the majority of the protoplast culture was to incubate it without agitation and with low light irradiation < 60 μmol photon m^−2^ s^−1^. Recovery trends were studied by analyzing the growth of microalgae cells every two days via optical measurement (OD_680 nm_) for a time course of 12 or 15 days in total. This interval was chosen because the green growth stage in *H. pluvialis* usually lasts from 9 to 20 days based on the relationship between cell biomass and cell activity [23].

**Figure 2:**
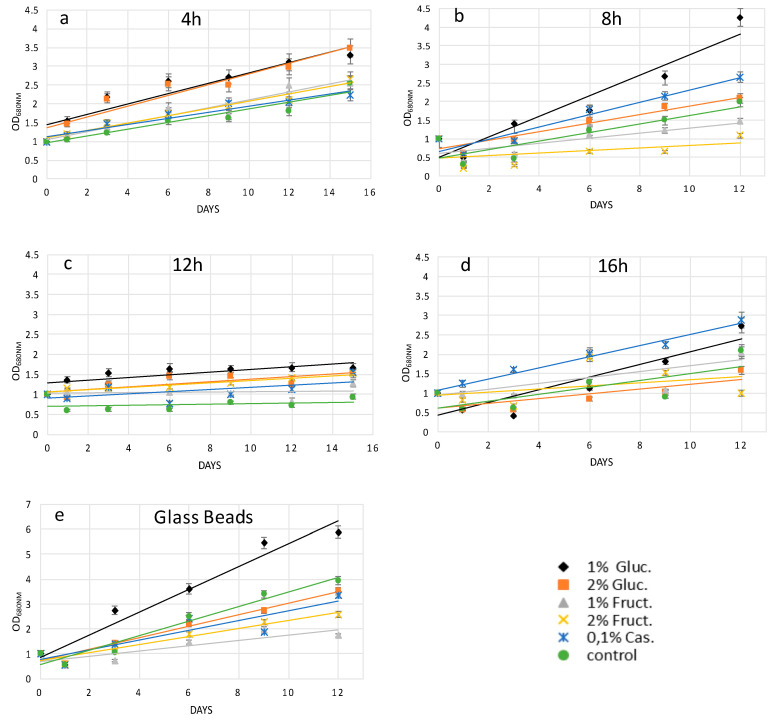
Recovery trend lines of *H. pluvialis* cells before and after enzymatic/mechanical treatment in media supplemented with an organic source of carbon (**a**) after 4 h of enzymatic hydrolysis; (**b**) after 8 h of enzymatic hydrolysis; (**c**) after 12 h of enzymatic hydrolysis; (**d**) after 16 h of enzymatic hydrolysis; (**e**) after mechanical treatment with glass beads. G.B. glass beads.

Following the 4-h cellulase treatment, protoplast cells grown in supplemented media showed a different recovery trend as compared to protoplasts grown on the medium without any additions (control). Here, 1% and 2% *w*/*v* glucose showed better growth, and probably better wall regeneration, while 1% and 2% *w*/*v* fructose scored as the second more efficient group of carbon compounds, with casamino acids being the least effective after 15 days of growth (Figure 2a). The effects of prolonged digestion started to be evident after 8 h of cellulase treatment, since 1% and 2% *w*/*v* fructose impaired the recovery trend of *H. pluvialis* protoplasts. Indeed, both fructose concentrations performed worse than the control and the glucose-supplemented media. Casamino acids enhanced the recovery performance of protoplasts as compared to 2% glucose but not as compared to 1% glucose. Therefore, after 8 h of digestion, 1% glucose was confirmed to be the best supplement for a fast recovery (Figure 2b).

After 12 h of cellulase treatment, all the extra added sources enhanced the recovery of *H. pluvialis* protoplasts in comparison to protoplasts grown in control medium. Here, 1% fructose showed the lowest recovery efficiency followed by 0.1% casamino acids. Next, 2% *w*/*v* glucose and 2% *w*/*v* fructose sufficiently enhanced growth and regeneration; however, the highest efficiency was obtained with 1% *w*/*v* glucose, again being the key concentration and compound for the best performance after 15 days of growth (Figure 2c).

Casamino acids were the crucial compound for the best recovery of *H. pluvialis* protoplasts after the longest cellulase treatment of 16 h. Higher concentrations (2% *w*/*v*) of glucose and fructose did not enhance protoplasts regeneration with respect to control growth medium. Lower concentrations (1% *w*/*v*) of glucose and fructose were more efficient, with glucose showing a higher recovery degree after 12 days of growth (Figure 2d). 

*H. pluvialis* protoplasts produced with the glass bead vortexing process in general showed higher recovery performance in all five supplemented media as compared to chemical treatment. However, glucose at concentrations of 1% *w*/*v* was confirmed to be the key compound for the recovery. Fructose and casamino acids did not improve the protoplast growth as compared to medium without any carbon or amino acid source addition (Figure 2e).

**Figure 3:**
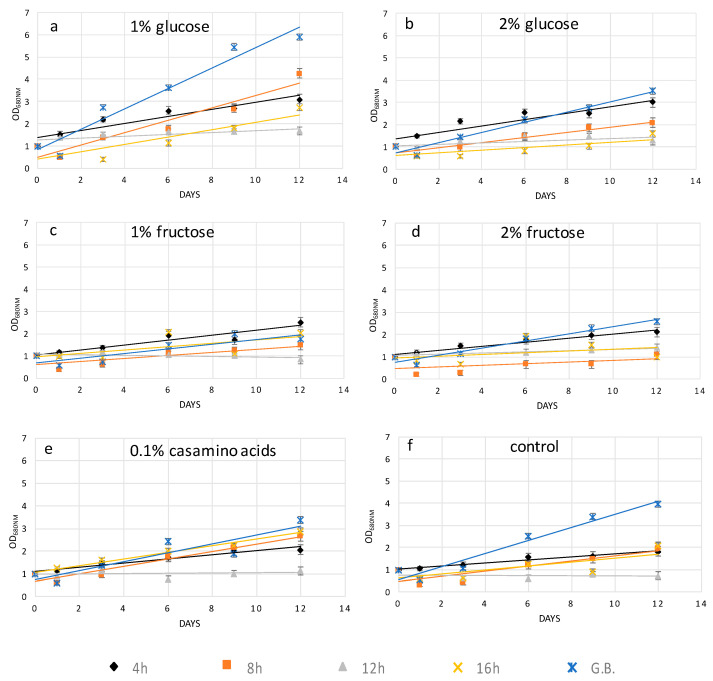
Recovery trend lines of *H. pluvialis* cells before and after enzymatic (4, 8, 12, 16 h)/mechanical treatment in media supplemented with (**a**) 1% glucose; (**b**) 2% glucose; (**c**) 1% fructose; (**d**) 2% fructose; (**e**) 0.1% casamino acids; and (**f**) control.

From the above results, it was clear that the recovery rates strongly depended on the recovery media. To highlight this point, the protoplast growth rate was plotted versus organic carbon or nitrogen sources. From Figure 3, it can be seen that 1% and 2% glucose gave the best results as the OD range was between 0–6 and 0–3.5, respectively. Furthermore, *H. pluvialis* protoplasts prepared with the glass beads treatment and then recovered with 1% glucose medium represent the best protocol to have a high quantity of transformable cells with the highest recovery. 

### 2.3. Transformation of H. pluvialis Protoplasts Using Iron Oxide Magnetic Nanoparticles

To test the quality of protoplasts prepared as described above as well as the recovery protocols, we performed transformation experiments that may simulate the biotechnological applications of *H. pluvialis*. 

**Figure 4:**
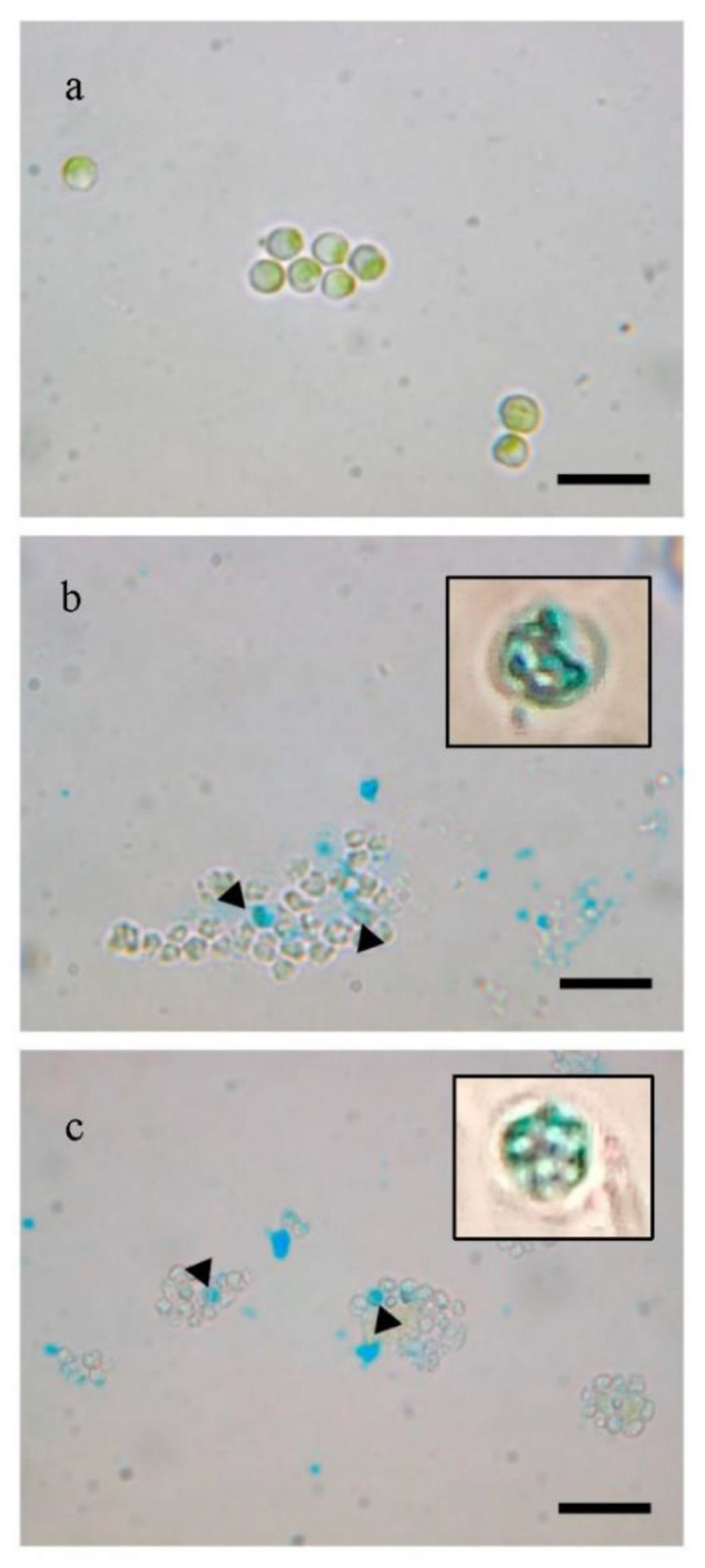
Visualization of the internalized magnetic iron oxide nanoparticles by light microscopy after Prussian blue staining. *H. pluvialis* cells were cultivated for 3 days after electroporation. (**a**) *H. pluvialis* untreated cells from stock culture, (**b**) *H. pluvialis* cells pretreated with enzyme for 8 h, (**c**) *H. pluvialis* cells pretreated with glass beads. In (**b**,**c**), a magnification of two cells stained in blue is reported, a digital zoon was used. Scale bar 10 µm.

The successful manipulation of *H. pluvialis* protoplasts with enzymatic or mechanical treatment was tested by inserting magnetic nanoparticles using electroporation. Due to the high volume (>20 mL) of each protoplast preparation, in order to allow the magnetic nanoparticles to enter the protoplast cells, a homemade flow-cuvette that works with standard electroporators was used. The characteristics of the flow-cuvette will be published elsewhere [24], but a brief description is given in the Section 4. Biocompatible iron oxide (Fe_3_O_4_) nanoparticles at a concentration of 10 ng/mL were used to transform *H. pluvialis* protoplasts via electroporation. The intracellular introduction of nanoparticles in both cases of pretreatment was confirmed by light microscopy images upon Prussian blue staining. The staining is specific for the detection of the iron ions. As it is depicted in Figure 4, all cells containing iron in their cytoplasm appear to be blue.

**Figure 5:**
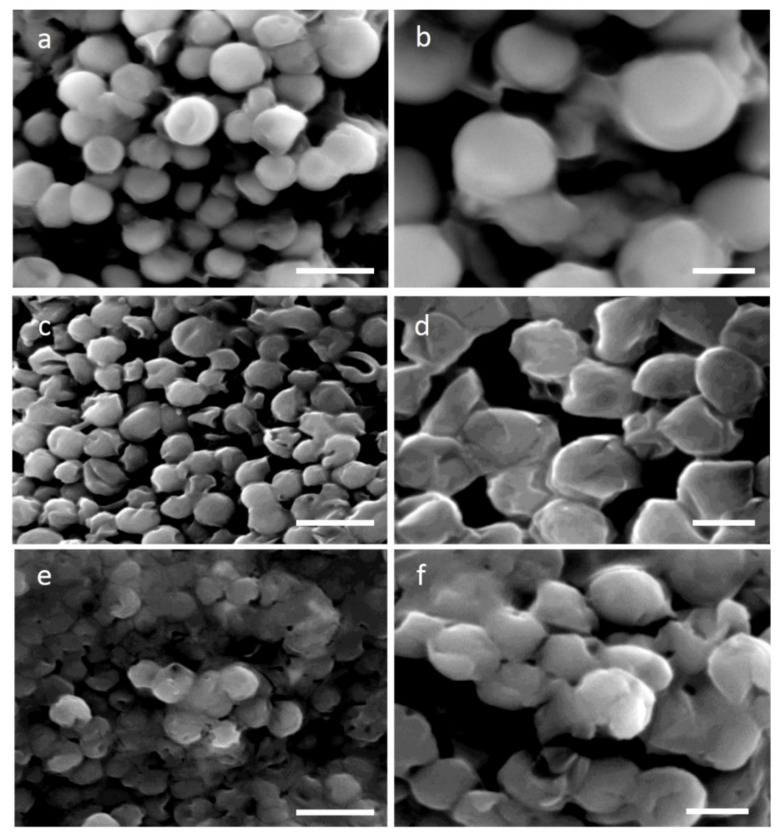
Recovery of *H. pluvialis* after the 8-h enzymatic digestion and the glass bead treatment as well as their transformation with magnetic particles. (**a**,**b**), two different magnifications of *H. pluvialis* control cells. (**c**,**d**), two different magnifications of *H. pluvialis* cells digested with cellulase and after 4 days of recovery. (**e**,**f**), two different magnifications of *H. pluvialis* cells pretreated with glass beads and after 4 days of recovery. Scale bar on the left column is 5 µm whereas in the right column, it is 2 µm.

**Figure 6:**
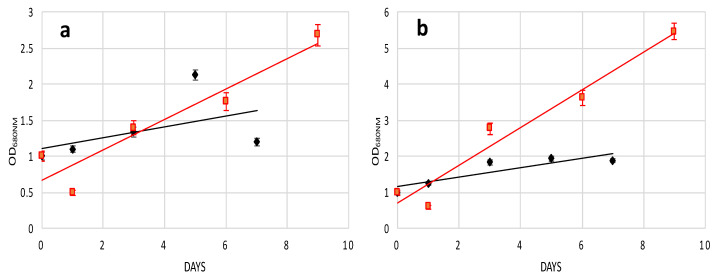
Recovery trend lines of *H. pluvialis* protoplast cells after transformation with magnetic nanoparticles. (**a**) *H. pluvialis* protoplast pretreated with cellulase digestion (8 h) in media supplemented with 1% glucose. (**b**) *H. pluvialis* protoplast pretreated with glass beads in media supplemented with 1% glucose. (**a**,**b**) Overlapping of recovery trend lines of *H. pluvialis* protoplast pre-treated with both methods, non-electroporated cells (red) and electroporated cells (black).

The recovery of *H. pluvialis* protoplasts following the 8-h digestion as well as their transformation with magnetic nanoparticles were visualized by SEM images (Figure 5c,d) after 4 days of culturing, compared to the control cells (Figure 5a,b). The analysis of the pictures demonstrated a similar shape between the untreated and treated/transformed cells, which coincides with the fact that 1% *w*/*v* glucose supported the regeneration of protoplasts. The successful recovery of transformed protoplasts was also verified by the recovery growth curve, which shows an increase of chlorophyll content up to 4 days (Figure 6a), whereas the absorbance started to decay at day 5, most probably due to the consumption of nutrients in the medium. The protoplast and electroporation efficiency was calculated (as described in the materials and methods) and the results are presented in Table 1. The best result gave 30% of magnetic cells in the preparation for glass bead treatment, while after enzymatic digestion, the efficiency was around 20%.

**Figure 7:**
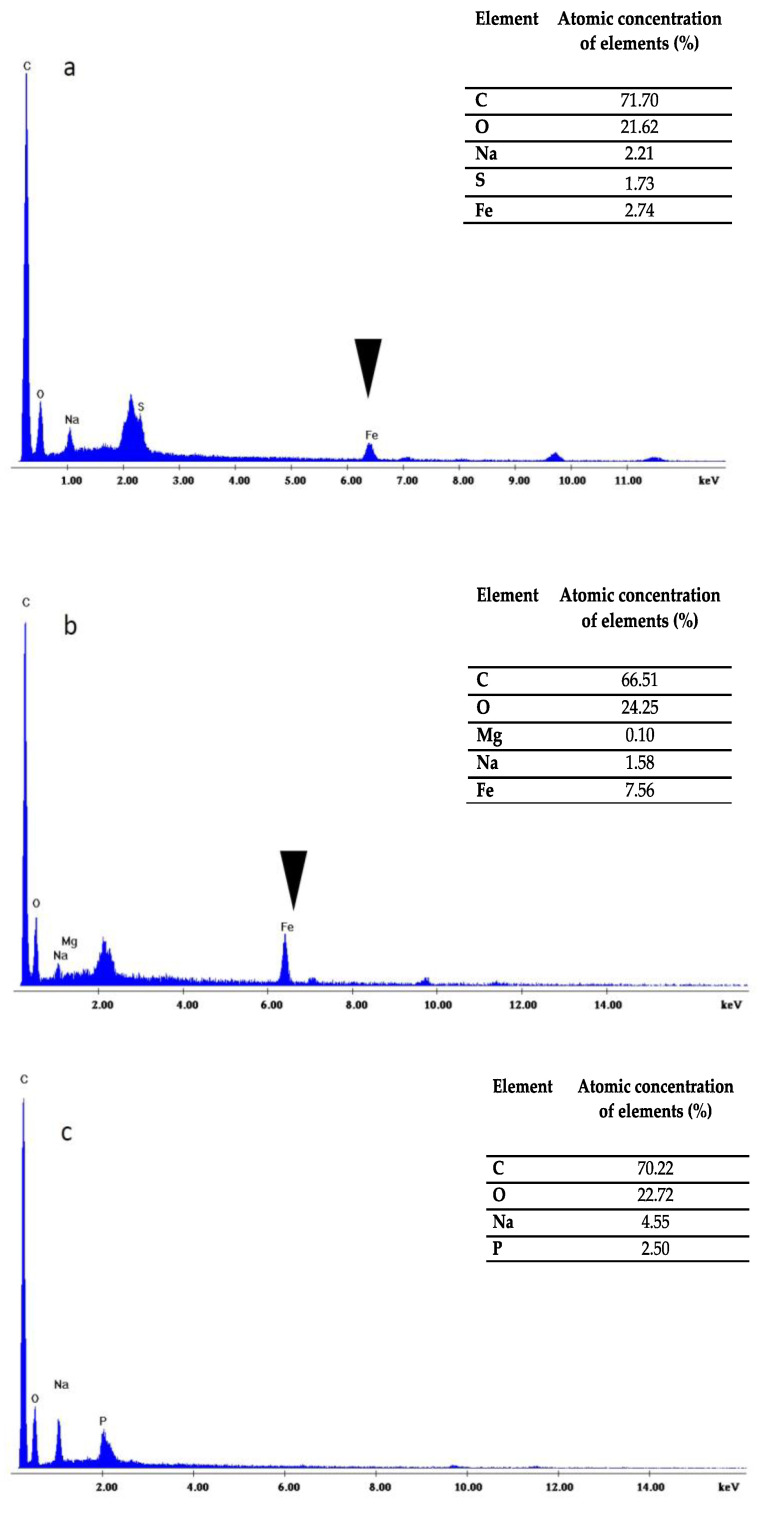
EDX analysis for the detection of the elemental composition of the substance. (**a**) EDX analysis of digested and transformed cells where the iron peak (arrow) is visible. (**b**) EDX analysis of glass bead-treated and transformed cells where the iron peak (arrow) is visible. (**c**) EDX analysis of control cells where the iron peak is not detected.

The SEM micrographs also revealed that after 4–5 days, the cells were able to fully recover despite their suffering due to the two stressing insults (pretreatment and electroporation). Indeed, probably because of the presence of organic carbon, they were able to reconstruct their cell wall (Figure 5c,d). Moreover, the chemical distribution analysis (EDX) showed the presence of iron (Figure 7a) only in the cells transformed with the magnetic nanoparticles but not in the control cells (Figure 7c), thus confirming the Prussian blue data.

The recovery of *H. pluvialis* protoplasts after the glass bead vortexing and their transformation with magnetic nanoparticles was also studied. It was found that, similarly to the protoplasts prepared with the enzymatic digestion, the cells were able to sufficiently reconstruct their wall after 4–5 days of cultivation in the recovery medium (Figure 5e,f). The EDX analysis performed on this sample also confirmed the presence of iron in the transformed cells (Figure 7b) whereas the recovery of the culture was efficient after 3–4 days. Indeed, after 5 days, the recovery curve showed a decay similar to the one obtained with enzymatic digestion (Figure 6b), since the culture reached its saturation point and re-culture in fresh medium was necessary. Furthermore, to verify if the electric shock suffered by protoplast cells could have effects on their recovery, we overlapped the trend lines of Figure 6a,b with the corresponding trend lines of non-electroporated cells of Figure 2. As it can be seen from Figure 6a,b, the electric shock affected the recovery of protoplast cells. Up to the fifth day of recovery, the trend points were very similar; after, media for the electroporated cells started to decay and in turn impaired the cell growth. 

## 3. Discussion

Biotechnological or genetic transformation of microalgae cells could be succeeded through a suitable cell wall perturbation, which permits the insertion of DNA/RNA or nanoparticles and simultaneously supports the normal regeneration and growth of microalgae. Microalgae cells, like bacteria, present a rigid cell wall that surrounds the plasma membrane. Besides structural properties, the cell wall is a formidable barrier that does not allow the insertion of macromolecules into the cytoplasm, even by forcing them with methodologies like electroporation. Indeed, we tried to transform microalgae cells with magnetic nanoparticles, but the rate of magnetic cells was nearly zero. Therefore, we opted to transform protoplast. Protoplasts obtained from terrestrial or aquatic photosynthetic organisms find a variety of applications both in basic and applied research. They can be prepared from multicellular plant tissues or simply by removing the cell wall from unicellular photosynthetic microorganisms, such as microalgae. Recent studies have verified that protoplasts derived from various tissues have crucial differences and properties, especially those that are genetically or biotechnologically transformed [16]. Depending on the final application, the degree of cell wall removal might vary. Indeed, for cell fusion, perfectly “naked” protoplasts are required since the presence of any rigid cell walls may hamper the process, reducing its efficiency and yield. Fused microalgae (*Ochromonas danica* and *Haematococcus pluvialis*) protoplasts via PEG treatment resulted in enhanced fatty acid production, while *Chlorella kessleri* and rat insulinoma cell line fusion created insulin-producing cells [25,26]. On the other hand, when protoplasts are intended to be used for genetic manipulation, e.g., transfection with plasmid, siRNA, CRISPR, and others, removal of the entire cell wall might not be mandatory since the intracellular delivery of such macromolecules can be forced with appropriate methodologies. Other important parameters to be considered in protoplasts’ preparation are the time needed for the recovery (cell wall reconstruction), incubation temperature, means of transfection, number of protoplasts cells required (biomass), and cost of protoplast preparation. These parameters are crucial especially when protoplasts are used in plant/algae industrial applications where the economic viability and sustainability of the entire production process is carefully evaluated.

In this work, we reported how to weaken the *H. pluvialis* cell wall, through single-enzyme digestion and mechanical processes as well as their regeneration, in order to produce large quantities of transformable protoplasts, for use in several fields of blue-biotechnology. All experiments were performed with relatively high volumes, 20–100 mL, which can be easily scaled up, and cost minimization related to the enzymes and energy utilized. The *H. pluvialis* cell wall is constituted by 19% of carbohydrates and 75% of proteins in the exponential phase of growth, while at the stationary phase of growth, 70% of carbohydrates (66% hexoses and mannose), 3% cellulose, and 6% proteins, as well as a remaining 3% of acetolysis-resistant material, are crucial components of the cell wall [14]. Cells of this strain can be categorized into flagellates and resting cells. Several enzymes have been reported to be used in protoplasts formation from microalgae, such as proteinase K, a mixture of cellulase, hemicellulase, pectinase, sulfatase, tyrosinase, and others, with good results [12,27,28,29,30,31,32,33,34]. We sought to investigate the possibility of using a single-enzyme digestion with cellulase for the minimum digestion time in order to minimize cost and reduce the time of *H. pluvialis* protoplast production. In our study, the usage of cellulase, which catabolizes cellulose as a linear polymer of D-glucose with β-1,4 linkages, decomposed the cell wall almost to the same efficiency at different treatment periods between 4 and 16 h as it was shown by the SEM images (Figure 1a–f). To identify the minimum digestion time using only cellulase as the hydrolytic enzyme at a concentration of 2% (*w*/*v*), we performed a series of digestions with incubation of 4, 8, 12, and 16 h. After each incubation, the entity of cell wall damage was studied by SEM, as well as the transfectability by introducing, via electroporation, magnetic nanoparticles with a diameter of 100 nm. Analyzing the SEM images, we did not observe significant differences between 4 and 16 h of digestion time, as shown in Figure 1c,d,e,f, though a good transfection outcome was obtained with cells digested for at least 8 h.

We also noticed that morphologically, the digestion time did not widely affect the cell shape (Figure 1). The fact that after 16 h, digestion protoplasts presented almost the same appearance could be due to the low percentage of cellulose in the cell wall. Indeed, as mentioned above, cellulose represents only 3% of the cell wall, but it plays an important role in the cell wall structure since cellulose microfibrils offer support for sugar-based scaffolds, such as hemicellulose, proteins, and other macromolecules. Thus, even though it is not visible, a long hydrolysis of cellulose microfibrils may have a significant effect on the permeability of the cell wall to macromolecules as well as certain nanomaterials. In addition, it has to be considered that different protoplast yield variations can be due to physiological and biochemical conditions and even to be related to the culture ageing [27] while different enzyme batches may affect the yield between the same species. 

A second parameter we tried to optimize was the incubation temperature. In the literature, incubation temperatures of 20–25 °C have been reported as the optimal temperatures for species like *Gracilaria* and *Ulva*, while an increased temperature may decrease their protoplast formation productivity [30,33]. Regarding our experiments with *H. pluvialis*, an incubation temperature of 30 °C resulted in optimal cell wall digestion, which confirms the similar performance obtained in *Chlorella* species [35]. 

Mechanical treatment through glass bead vortexing resulted in a higher efficiency of cell wall disruption of *H. pluvialis* cells treated, as this was verified by the SEM images (Figure 1g,h). Microalgae cells mixed with glass beads and vortexed for 30 s, while a prolonged vortexing time resulted in irreversible damage, which caused cell death. In *Chlamydomonas* species, 15 s demonstrates a better recovery than 60 s of glass bead vortexing [36]. Studies revealed that the pretreatment of the cells upon acidic or thermal conditions may reduce the energy need for mechanical rupture of microalgae cells [15], while allantoin pretreatment for *Porphyra* cells enhanced the protoplasts formation [37]. The method we proposed to produce protoplasts seems to better in terms of cost and time, since there is no need for sharp control of the incubation temperature, while the glass beads can be reused multiple times after proper cleaning.

Independently of the protoplast preparation method used, the cells undergo stressing conditions, which may lead to their death if not properly recovered. Cell wall regeneration is an important step in order to have fully exploitable protoplasts cells able to be properly grown. In this work, in order to facilitate cell wall reconstruction, the growth medium was supplemented with an organic source of carbon or nitrogen. Indeed, it was noticed that protoplast cells, especially during the initial days after their preparation, had a reduced photosynthetic ability and most probably were not able to produce an adequate quantity of glucose necessary for all biological processes. It was also observed that high light and agitation time negatively affected the protoplast culture viability (data not shown). Glucose at 1% *w*/*v* was the crucial compound, which enhanced the recovery trend of *H. pluvialis* protoplasts upon the mechanical process and enzymatic digestion after 4, 8, and 12 h of treatment, while casamino acids at 0.1% *w*/*v* better supported protoplast renewal after 16 h of cellulase treatment, as depicted in Figure 2a–e. The different key compounds used for protoplast recovery imply the activation of different metabolic pathways, which are related to the degree of cell wall degradation. For instance, glucose in comparison to fructose as the initial compound of glycolysis may lead to enhanced production of cellulose. In conclusion, glucose as the key metabolite for glycolysis enhanced the recovery trends of *H. pluvialis* protoplasts after the short-term cellulase treatment and glass bead agitation, while amino acids more efficiently promoted protoplast regeneration after the enzymatic treatment.

An important parameter that has to be considered in the protoplast preparation from microalgae is the osmotic fluctuation, since they live in salty or fresh water, where electrolytes, ions, and other molecules present affect the osmotic pressure in the inner cell compartment. Therefore, by removing the protective cell wall, microalgae could undergo damage since water can spontaneously enter the cell body and inflate it until its explosion. To prevent such a phenomenon, during protoplast preparation and in all downstream applications, sorbitol and mannitol are added in the medium. These two sugar-based molecules increase the osmotic pressure of media by equilibrating the pressure between the internal cell compartment and the external environment. The optimized concentration of mannitol is 0.6 M, and it is used in all media during digestion, electroporation, and recovery along the medium specific for *H. pluvialis*.

Finally, the quality of the protoplasts prepared with both methods was tested via their transformation with magnetic nanoparticles. The presence of the magnetic particles inside microalgae cells was easily verified by means of magnetism and by the Prussian blue test. Our results showed that with enzymatic digestion and glass bead pre-treatment, the transformation efficiency with magnetic particles was around 20% and 30%, respectively. It is worth noting that when Prussian blue staining was negative (no blue color overlapped with green), the attraction of the cells with permanent magnets was not possible, suggesting that only when nanoparticles were internalized the cells were magnetic. The transformation yield after cellulase treatment was lower compared to plant protoplasts transformed with iron nanoparticles due to the double-enzymatic effect of cellulose and macerozyme R10 compared to our single-enzymatic treatment [38], while similar results to ours were demonstrated in aminoclay transformation with vortexing of *Chlamydomonas reinhardtii* [39].

In conclusion, our study demonstrated a simplified methodology to obtain good-quality *H. pluvialis* protoplasts. Glucose should be added to the recovery medium as the key metabolite for enhancement of the recovery trends of the protoplasts after short-term cellulase treatment or glass bead agitation, while amino acids more efficiently promote protoplast regeneration after the enzymatic process. Osmotic stabilizers are necessary in all steps, from pretreatment to recovery. By following our study, it will be possible to easily prepare a large quantity of *H. pluvialis* protoplasts biomass, transform them with proper molecules in order to acquire magnetic properties, and recover them after 4–5 days of growth with conditional media.

## 4. Materials and Methods

### 4.1. Organism and Growth Conditions

The *H. pluvialis* strain (CCAP 34/6) was purchased from Culture Collection of Algae and Protozoa (Dunbeg, UK). The strain was grown in medium with the following composition (per liter): Ca(NO_3_)_2_, 0.15 g; KNO_3_, 0.10 g; β-glycerophosphoric acid disodium salt pentahydrate, 0.05 g; MgSO_4_·7H_2_O, 0.04 g; Tris-aminomethane, 0.50 g; thiamine, 0.01 mg; PIV metal solution, 3.00 mL; biotin 0.10 μg; and vitamin B_12_, 0.10 μg. One liter of PIV metal solution contained Na_2_EDTA, 1.0 g; FeCl_3_·6H_2_O, 0.196 g; MnCl_2_·4H_2_O, 36.0 mg; ZnSO_4_·7H_2_O, 22.0 mg; CoCl_2_·6H_2_O, 4.0 mg; and Na_2_MoO_4_·2H_2_O, 2.5 mg [40]. The pH was adjusted to 7.5 and the temperature was controlled at 23 °C. Cell cultures of *H. pluvialis* were grown in 250-mL Erlenmeyer flasks containing a culture volume of 100 mL at 150 rpm supplemented with NaHCO_3_ at a concentration of 1 g/L. The re-culture concentration was adapted to an optical density of 0.1 at 680_nm_ (OD_680 nm_). Periodic purity assessment was performed by microscopic examination. The photobioreactor was illuminated at 60 μmol photon m^−2^ s^−1^ 24/7 white LED lamps at the cell culture surface. *H. pluvialis* growth was monitored by measuring the OD at 680 nm with a UV-vis spectrophotometer (Shimadzu, Kyoto, Japan) for up to 15 days. All experiments were conducted in duplicate while samples were analyzed in triplicate.

### 4.2. Cell Wall Disruption by Enzymatic Lyses

*H. pluvialis* cells were grown in a 500-mL flask for 6–8 days and harvested by centrifugation at 1000× *g* for 5 min. Then, the cell pellet was suspended in 25 mM phosphate buffer (pH 7.0) containing 0.6 M D-mannitol and the polysaccharide-degrading enzyme cellulase (2%). The incubation temperature was chosen based on the literature as well as our preliminary data, where different incubation temperatures (25–37 °C) were examined and 30 °C was found to give the best results based on our morphological observations via SEM. Each reaction mixture was incubated at 30 °C for 4, 8, 12, and 16 h. Afterwards, the cells were centrifuged at 1000× *g* for 3 min, the pellet was re-suspended in 0.6 M D-mannitol solution, and was washed twice with sugar solution. The protoplast layer was transferred into 5 mL of buffer (pH 7.0) containing 0.6 M D-mannitol solution for further analysis. Generally, cells were grown up to an optical density of 0.8–1.0 at 680 nm. Then, from this cultivation, 100 mL of cells were withdrawn, washed, and further processed. The digestion was performed in a final volume of 30 mL. After digestion, enzymes and digestion debris were removed by 2 washing steps and cells resuspended in 20 mL of medium for electroporation. All solutions used for digesting, washing, and electroporation contained 0.6 M D-mannitol to equilibrate the osmotic pressure.

### 4.3. Cell Wall Disruption with Glass Beads

Glass beads (Sigma, St Louis, MO, USA), 1.0 mm in diameter, were washed in concentrated sulfuric acid, then rinsed thoroughly with sterilized water a few times, and baked at 180 °C for 2–3 h. *H. pluvialis* cells (from the same culture as before) were cultured to the logarithmic phase and harvested by centrifugation at 1000× *g* for 5 min. Cells were washed three times with 25 mM phosphate buffer (pH 7.0) containing 0.6 M D-mannitol and re-suspended with this medium at a concentration of 2 × 10^8^ cells/mL. A total of 200 mg of dry glass beads and 1 mL of cells (2 × 10^8^ cells/mL) were added to a 1.5-mL Eppendorf tube and agitated at 1500 rpm on a vortex agitator for 30 s. The cells were transferred to sterilized test tubes for further analysis. The experiments were performed with the optimum conditions for 30 s and 200 mg of dry glass beads. Blank controls were transformed without agitation. Every treatment consisted of three independent agitations.

### 4.4. Morphological Observation with Scanning Electron Microscopy Coupled with an Energy Dispersive X-ray Analyzer System (SEM-EDX)

The *H. pluvialis* algal cells were sampled for the scanning electron microscopy observations immediately after cell disruption by enzymatic lyses as well as mechanical treatment. The samples of algal cells were fixed in 2.5% (*v*/*v*) paraformaldehyde at 4 °C for 1 h, rinsed three times in 0.05 M phosphate buffer (pH 7.4), and successively dehydrated in 20%, 35%, 50%, 75%, 90%, and 100% (*v*/*v*) ethanol solution for 5 min. After dehydration, the prepared samples were sputter-coated with Au and examined using a FEI Quanta-200 Scanning Electron Microscope. For validation purposes, magnifications of 5000× and 10,000× were used. The microscope was equipped with an EDX detector in order to obtain a distribution of the elemental composition of cells. The *X*-ray spectrum of each sample loaded with a given microelement was obtained.

### 4.5. Nanoparticles

For the experimental investigations, ferrofluid nanoparticles (fluidMAG-lipid from Chemicell GmbH, Berlin, Germany) were used. The nanoparticles consisted of an aqueous dispersion of magnetic iron oxides (diameter of 80–100 nm) and were covered with phosphatidylcholine. The surfactant, phosphatidylcholine, over the iron oxide core, is a coating layer similar to cell membrane. This characteristic gives it an excellent biocompatibility since coated nanoparticles do not cross react with molecules and organelles of the cytosol.

### 4.6. Protoplast Transformation

The transformation of *H. pluvialis* protoplasts with magnetic nanoparticles was performed using a homemade continuous flow cuvette device for the electroporation. Pretreated *H. pluvialis* cells were dissolved in medium containing 0.6 M D-mannitol solution and electroporated with a pulse generator (MicroPulser) from BioRad (USA) using 1 pulse at 3 kV. Nanoparticles and pretreated cells were mixed for 30 min before the electric pulse. A homemade flow-cuvette was used based on 3-D printing of a plastic core where aluminum plaques were glued. The internal volume of the flow cuvette was 1.5 mL with inlet and outlet tubes. A peristaltic pump was used to pump the mixture of protoplast cells and nanoparticles inside the flow cuvette at a rate of ~100 µL per s. The generator was modified with an external electronic circuit in order to deliver a pulse every 15 s.

### 4.7. Prussian Blue Staining

Cells were fixed with methanol/acetone 7:1 solution for 10 min at room temperature and washed twice with phosphate-buffered saline (Sigma, St Louis, MO, USA). Cells were incubated for 1 h in Prussian blue reagent obtained following the instruction manual of Biopal protocol (BioPhysics Assay Laboratory, Worcester, MA, USA). Stained cells were investigated using the microscope.

### 4.8. Regeneration of Protoplasts

To regenerate protoplasts, an efficient protocol was established for cell wall regeneration and restoration of the original *H. pluvialis* morphology. The *H. pluvialis* protoplast suspension was obtained as described above after cell disruption by enzymatic lyses and glass beads. The regeneration medium was supplemented with mannitol for osmotic stabilization of protoplasts [41], the growth medium, and different concentrations of glucose (1 and 2% *w*/*v*), fructose (1 and 2% *w*/*v*), and casamino acids (0.01% *w*/*v*) to enhance cell wall regeneration and subsequent division. In total, 10 mL of the suspension were placed with 90 mL of regeneration medium. Medium without a supplemented carbon or nitrogen source was used as a control. Protoplasts were incubated at 23 °C, without agitation and with low light intensity 60 μmol photon m^−2^ s^−1^ for about a week and regenerated protoplasts were observed every 3 days. The culture conditions were described above. The electroporation efficiency was calculated as follows: electroporated cells were grown in the appropriate recovery medium for 4 days, then the OD_680 nm_ was measured using 1 mL of culture. The same 1 mL was placed in a magnetic rack to attract and retain magnetic cells. After 20 min, the supernatant of the tube was carefully removed and its absorbance (OD_680 nm_) was measured again. When both protoplast preparation and electroporation were successful, a pellet of magnetic cells in the tube formed; therefore, the supernatant’s absorbance was lower compared to the initial one. The protoplast and electroporation efficiency was calculated by the rate between the two measurements.

## Figures and Tables

**Table 1:** Transformation efficiency of *H. pluvialis* protoplasts.

Conditions	OD_680 nm_ (4th Day after Recovery)	OD_680 nm_ (after Magnetic Rack)	Protoplast and Electroporation Efficiency (%)
Enzymatic digestion (8 h)	0.923	0.736	20.26
Pre-treatment with glass beads	0.889	0.625	29.7

Values are the means of three measurements and the standard deviation was below 5% in all cases.
